# Influence of Horse Age, Marinating Substances, and Frozen Storage on Horse Meat Quality

**DOI:** 10.3390/ani11092666

**Published:** 2021-09-10

**Authors:** Renata Stanisławczyk, Mariusz Rudy, Marian Gil, Paulina Duma-Kocan, Jagoda Żurek

**Affiliations:** Department of Agricultural Processing and Commodity Science, Institute of Food and Nutrition Technology, College of Natural Sciences, University of Rzeszow, St. Zelwerowicza 4, 35-601 Rzeszów, Poland; mrudy@ur.edu.pl (M.R.); mgil@ur.edu.pl (M.G.); pduma@ur.edu.pl (P.D.-K.); jagodazurek@yahoo.com (J.Ż.)

**Keywords:** horse meat, marinating, frozen storage, phosphates, organic acids

## Abstract

**Simple Summary:**

The age of horses can influence several properties of the obtained raw material. As the age of horses increases, the meat retains less water, and more fat (*p* < 0.05) and minerals. In general, horse meat from older animals exhibits undesirable stringiness and hardness, due to a large proportion of connective tissue (collagen). Currently, many methods are applied to improve the tenderness of meat. Of these, the most popular is marinating the meat with various substances, which enhances the functional and sensory properties of the meat. Freezing is a widely accepted method for extending the shelf life of meat. Both the technique used for freezing and further storage at negative temperatures have an impact on some of the properties of meat. Most importantly, the pH value, color, and water absorption of meat tend to change with freezing. In addition, the dry matter content and tenderness of meat increase. This study aimed to analyze the impact of horse age, marinating substances, and frozen storage on the quality of horse meat. As horses age, the values of meat cutting force increase (*p* < 0.05). For example, the cutting force increases by 4.57 N/cm^2^ during the first period of freezer storage, and by 3.28 N/cm^2^ after 3 months of freezer storage (*p* < 0.05).

**Abstract:**

The present study analyzed the influence of horse age, substances used for marinating, and frozen storage on the quality of horse meat. It was conducted on the samples of the longest thoracic muscle, obtained from 12 carcasses of horses (aged 4–7 and 8–12 years). Among the analyzed samples, a higher fat content (*p* < 0.05) was found in the meat obtained from the carcasses of older horses. The pH value of the meat samples was influenced by the treatment applied (*p* < 0.05). Of the substances used for marinating, malic acid caused a decrease in the pH of the meat obtained from young horses (*p* < 0.05). A similar effect was observed with the addition of phosphates to malic acid-marinated meat. On the other hand, the use of phosphates for marinating resulted in an increase in the pH of the meat obtained from older horses (*p* < 0.05). The substances used for marinating the horse meat did not significantly affect the reduction in cutting force values. Furthermore, the values of shear force, hardness, stiffness, gumminess, and chewiness of the meat increased with horse age (*p* < 0.05). An influence on the color parameters a* and b* of the meat was found for the interaction between age, storage period, and the type of treatment (*p* < 0.05). The use of lactic acid and malic acid for marinating the meat of young horses caused a decrease in the proportion of red color (4.67 and 3.43) and an increase in the proportion of yellow color (3.81 and 1.71), especially after 3 months of freezer storage. All the substances used for marinating (except for phosphates) were associated with higher (*p* < 0.05) thermal and forced drips of meat from the carcasses of both young and older horses during each storage period, in comparison to the control. The interaction between age and the type of treatment had an influence on the tenderness and juiciness of the horse meat (*p* < 0.05). In sensory evaluation, it was noted that the interaction between age and the treatment procedure influenced the tenderness and juiciness of the meat samples (*p* < 0.05). There is still a need for further research to increase knowledge regarding how to improve the quality of horse meat, and ultimately increase the demand from consumers and meat processing plants.

## 1. Introduction

Horse meat is a distinct food with a specific consumer base, and its production is very popular all over the world. Young, well-muscled animals, whose meat is highly valued and willingly bought, are targeted for slaughter. In Europe, horse meat is mainly consumed by Italians, followed by Belgians, with an annual consumption per capita of 0.88 and about 0.5 kg, respectively [1]. Horse meat is particularly popular in Western European countries, where it is treated as an equivalent to other meat types and is often valued higher than beef or pork. In Poland, horse meat is not consumed, due to various reasons, including emotional resistance, lack of skills in preparing horse meat dishes, and a traditional consumption model that prefers other types of meat. Popularizing horse meat in Poland also requires breaking traditions regarding its distribution. Horse meat is incorrectly regarded as unworthy of consumption and promotion, and it is worthwhile to make attempts to popularize its consumption, since horse meat constitutes a significant reserve of meat mass that can be utilized. Moreover, there are very few promotional activities promoting the nutritional value of horse meat and the products made from it, and due to their high price, almost all products are sold to richer EU countries.

The low popularity of horse meat in Poland is also associated with its poor quality. In the past, it was mainly obtained from older animals, which are not suitable for export. Horse meat from older animals is characterized by low tenderness, considerable hardness and coarseness, a very dark color, and a high fat content [2]. In 2019, horse production in Poland was estimated at 27 thousand heads and horses, with a total weight of 13.3 thousand tons (industrial slaughtering of animals) [3]. Due to the fact that horse meat is rarely consumed in Poland, 80–95% of domestic production is exported. The main customers are Italy, where draft horse meat is highly valued, followed by France, Belgium, Austria, and Germany, which account for 70–72% of horse meat export from Poland [2]. Horse meat is a source of valuable nutrients. It is lean, has a low fat content [4,5,6,7,8], and is rich in proteins with a high biological value as well as desirable amino acids [9,10,11]. However, unlike the meat obtained from other animals, horse meat has a high amount of glycogen, which imparts a sweet taste [12,13]. Another unfavorable quality of horse meat is its dark red color, accompanied by a faint brown tinge, due to the high concentration of the muscle pigment myoglobin [9,14,15,16,17]. In addition, the meat darkens with the age of the animal, while the fat turns yellowish or even orange in color. Horse meat also has a large proportion of connective tissue (collagen), which is an additional distinguishing feature [16,18,19]. The age of animals is considered an important influencing factor of the obtained raw material, because as the animal gets older, several changes occur not only in the chemical composition and color of the meat, but also in the structure of proteins in the muscle and connective tissue. The mechanical stability of connective tissue increases with age, due to the cross-linking of collagen. As horse meat has a high content of collagen, it undergoes softening and physicochemical transformations for a long time in an acidified environment [20,21]. Maturation is a critical step in the production of culinary horse meat, which should be conducted with care and for a sufficiently long duration. However, it is one of the least complicated treatments, and improves the acceptance and suitability of horse meat. Significant changes also take place in meat during storage, and the lower the storage temperature is, the smaller the changes are. Freezing is a commonly used method that maintains the quality and durability of perishable meat [22,23,24]. The changes in meat quality caused by freezing are determined by the technique used and the subsequent frozen storage. The post-slaughter maturation processes of meat are slowed down or inhibited as a result of freezing. On the other hand, the processes responsible for water freezing and the formation of ice crystals within the muscle structures are intensified. Although freezing is important for the preservation of meat, both freezing and further storage at negative temperatures directly affect some of its properties. Most importantly, the pH, color, and water absorption of meat tend to change, while the dry matter content and tenderness of meat increase. Furthermore, the size of thermal drip and gel ability of muscle proteins change after the thermal treatment. Freezing also results in loosening of the capillary structures of muscle tissue, which, in turn, leads to a reduction in the tissue’s ability to retain its water during defrosting, as well as significant losses during thermal treatment, thus affecting the juiciness of the meat [25].

Currently, the approach used to improve the functional and sensory properties of meat is marinating. This procedure involves soaking, injecting, or mixing the product with aqueous solutions containing various ingredients. A marinade is a water solution composed of salt and additional substances. The brine composition is selected individually for each product, taking into account the necessary additives [26,27].

Due to the specific effect on muscle proteins, phosphates added for marinating increase their water absorption, and improve the binding and emulsifying properties. They also enhance the textural properties and consistency of the product, stabilize the color and fat emulsion, and increase the production efficiency. In meat processing, phosphates mainly help to dissociate the actomyosin complex, regulate the pH of the product, and increase the ionic strength of the environment and complex divalent cations [28,29]. Polyphosphate-induced changes in the water absorption of meat, together with the electrostatic effect of phosphate ions on proteins, not only alter the conformation of protein molecules, but also change the structure of the surrounding water molecules. This, in turn, improves the ability of proteins to bind water and emulsify fat. The increase in water absorption and the reduction in drip, caused by polyphosphates, can be partially explained by the increase in the pH of the cell fluid in relation to the isoelectric point of proteins. However, the pH increase is determined by the amount and type of phosphate added. A high water-binding capacity improves the binding of the plaster and keeps its surface dry, thus indirectly contributing to the stabilization of color. In addition, the sticky juice seals the pores in the muscle tissue and prevents the penetration of oxygen. This explains the antioxidant properties of phosphates and their ability to stabilize the color of cured products. These properties of phosphates are further enhanced by their metal ion-chelating activity. Moreover, phosphates limit the growth of spoilage microbes [29,30,31].

Organic acids can influence the muscle fibers and connective tissue, and contribute to improving the tenderness of muscle tissue. For example, citric acid, a food acidifier, is commonly used in marinating, as it not only increases the water-holding capacity and tenderness of beef muscles, but also acts as a chelator and controls the pro-oxidative activity of metals [32,33,34]. However, this acid can lower the pH of the meat, resulting in an excessively acidic flavor, and hence can decrease consumer acceptance. For this reason, solutions with an acid concentration exceeding 0.15 M are not recommended for marinating [35]. Nonetheless, this issue can be overcome by initially reducing the pH of the muscles with citric acid, to change the texture, and then increasing the pH by adding, for example, sodium triphosphate to improve the organoleptic characteristics of the meat [36]. The acids commonly used for marinating in the meat industry are lactic acid, which acts as an antimicrobial agent [32], and acetic acid, which is known for its acidity, pH-lowering ability, and bacterial growth-inhibiting properties.

The quality of horse meat determines its technological and culinary value. The meat industry currently aims at using methods that will allow the negative features of horse meat to be eliminated, especially related to the color and tenderness of this raw material [2]. The culinary use of horse meat justifies the need for further research on its properties. Knowledge of the specificity of horse meat, and especially the influence of various factors on its properties, will allow the selection of the appropriate technological procedure, and this thus will allow optimal processing of the raw material and the desired characteristics to be achieved. This would also provide an opportunity to disseminate and popularize horse meat-derived products.

The broad range of horse age (from foals to horses over 20 years old) causes large differences in the obtained raw material, because as the animal ages, not only does the tissue composition of the carcass change, but also the functional and sensory properties of the meat change. Because meat obtained from the carcasses of young and old horses varies in its properties, it is advisable to investigate and identify which method used to improve the tenderness of horse meat will enhance its quality parameters. A few studies have comprehensively analyzed the influence of horse age, marinating substances, and period of frozen storage on the quality of horse meat. Those studies [17,37,38,39,40,41,42,43] contribute to our understanding of how to improve the quality of horse meat, and ultimately increase the demand from consumers and meat processing plants. Nevertheless, there is still a need for further research to increase knowledge regarding this topic.

Therefore, the aim of this study was to determine the texture parameters, color, and sensory characteristics of horse meat, based on the animals′ age, marinating substances, and freezing storage time.

## 2. Materials and Methods

### 2.1. Raw Material

The study was conducted on Longissimus thoracis muscle isolated from 12 half-carcasses of younger horses (4–7 years) and 12 half-carcasses of older horses (8–12 years) from right side. The average age of the horses in the 4- to 7-year group was 5.5 ± 0.5, and that in the 8- to 12-year group was 10.0 ± 0.5. The age of horses was determined from the purchase documentation. Their weight antemortem was 500–560 kg (530 ± 30). Average carcass weight was 345 ± 30 kg. The horses used in the study were Malopolski and Silesian breeds and were obtained from farmers from Southeastern Poland. They were grouped according to their gender, as follows: 50% were geldings and 50% were mares. Each age group consisted of six half-carcasses of females and six half-carcasses of males. The horses were normal and maintained in an extensive system. After transport, the animals were kept in separate pens in livestock warehouses for about 24 h while maintaining animal welfare and under the supervision of the appropriate veterinary services. All horses were slaughtered the same day, by stunning with a captive bolt pistol according to the current methodology applied in the meat industry. From each carcass, and 24 h after slaughter, two samples (1000 g each half-carcass) from the M. longissimus thoracic, at the 13th–14th thoracic vertebrae level, were obtained to determine age, marinating substances, and frozen storage effect on meat quality. The medial portion of the samples collected was located at the height of the 13th–14th thoracic vertebrae. Each sample was cleaned to remove external fat, connective tissue, and tendons, and seven steaks (3 cm thick approximately) were obtained (from each age group, as follows: 12 half-carcasses × 2 M. longissimus thoracis samples × 7 steaks = 168 steak samples). One of the steaks was used as control, while the others were treated, after 48 h postmortem, with the corresponding compound in 1% solutions at an amount of 10% with reference to the sample weight. Muscle samples (steaks) were injected with the following reagents: (i) lactic acid (2-hydroxypropanoic acid, 80%); (ii) malic acid (hydroxysuccinic acid, 99%); (iii) phosphates (Hamina S containing emulsifiers)—E 451 (pentasodium triphosphate and pentapotassium triphosphate), E 450 (disodium diphosphate, trisodium diphosphate, tetrasodium diphosphate, tetrapotassium diphosphate, dihydrogen diphosphate, and calcium diphosphate), E 452 (sodium polyphosphate, potassium polyphosphate, sodium calcium polyphosphate, and calcium polyphosphate), and E 339 (monosodium phosphate, disodium phosphate, and trisodium phosphate (TSP)) with NaCl (salt); (iv) solution containing phosphates (Hamina S containing the abovementioned emulsifiers) and rosemary (0.1% rosemary oil); (v) solution containing lactic acid (2-hydroxypropanoic acid, 80%) and phosphates (Hamina S containing the abovementioned emulsifiers); and (vi) solution containing malic acid (hydroxysuccinic acid, 99%) and phosphates (Hamina S containing the abovementioned emulsifiers).

Then, they were immediately marinated in various aqueous solutions of the compounds at 1% concentration in glass vessels (solution/sample ratio = 2:1). Later, the marinated meat samples as well as control samples were refrigerated (6 °C) for 72 h. After storage, the meat samples marinated with lactic acid and malic acid were treated with phosphates by injecting 1% solution (Hamina S containing the abovementioned emulsifiers) at an amount of 10% with reference to the sample weight. These samples were marinated again in aqueous solutions of 1% phosphates (Hamina S containing the abovementioned emulsifiers and refrigerated (6 °C) for 24 h) in glass vessels.

After marinating, the batches of meat samples were subjected to flow freezing in a freezing cabinet (Budget Line type; Hendi, Warsaw, Poland), after vacuum packing them in PA/PE bags. At the beginning of freezing, the average temperature of meat was around 4 °C. Freezing at −28 °C was carried out for approximately 3 h. After freezing, the samples were stored for 1 and 3 months at −22 °C. Following predetermined periods of frozen storage, the samples were moved to the laboratory for analyses. Before quality testing, the packed samples were thawed at ambient temperature, approximately 10 °C. Once the temperature inside the meat sample reached 0 °C, defrosting was stopped and analyses were carried out.

### 2.2. Analytical Methods

The following parameters were measured in horse meat to determine its quality: chemical composition (amount of water, protein and fat content), pH, color, hydration properties (forced, thermal, and thawing drip), shear force of raw meat, texture (hardness 1 and 2, stiffness up to 5 and 8 mm, adhesiveness, resilience, cohesiveness, gumminess, chewiness, and springiness), and sensory quality (aroma intensity and desirability, tenderness, juiciness, taste intensity and desirability, general acceptability).

The water content of the samples was determined in accordance with the PN-ISO, 1442:2000 standard [44].

The protein content of the samples was determined using the Kjeldahl method. For this, the content of nitrogen calculated in the samples was converted into protein, according to the PN-75/A-04018 standard [45].

The fat content of the samples was determined using the Soxhlet method in accordance with the PN-ISO, 1444:2000 standard [46].

The active acidity (pH) was determined in cooled meat samples using an OSH 12-01 electrode and a CPC-411 pH meter (ELMETRON, Zabrze, Poland) with an accuracy of up to 0.01. Before measuring the pH, the device was calibrated with buffers of pH 4 and 7.

The color of the meat samples was measured in their cross-section in the CIE L* a* b* system, using a HunterLab UltraScan PRO (HunterLab, Reston, VA, United States) electronic spectrophotometer (D65 light source, measuring head opening: 8 mm, white reference standard calibration: L*—99.18, a*—0.07, b*—0.05). Parameter L* denotes brightness (spatial vector), parameters a* and b* are the trichromaticity coordinates (positive a* values indicate red color, while negative values indicate green color; positive b* values indicate yellow color, while negative values indicate blue color).

For measuring the physicochemical parameters, such as thermal and forced drips, the meat samples were first minced twice in a laboratory wolf device (Hendi, Warsaw, Poland) using sieves (4 mm diameter). Then, they were thoroughly mixed and homogenized, and subjected to further analyses.

Thermal drip of the meat samples was determined as described by Janicki and Walczak [47]. Briefly, a finely ground meat sample (weighing 20 g) was transferred to a hygroscopic gauze and heated in a hot water bath (85 °C) for about 10 min. After heating, the sample was cooled down to 4 °C and reweighed. Thermal drip was estimated based on the change in the weight of the sample recorded before heat treatment and after cooling as follows:

(1) $$\mathrm{Td}\;(\%)=\frac{WI-WII}{WI}\;\times \;100\%$$

where Td refers to the rate of thermal drip (%), *WI* refers to the sample weight before heat treatment (g), and *WII* refers to the sample weight after cooling (g).

Forced drip of the meat samples was determined as described by Grau and Hamm [48]. Briefly, a minced meat sample (weighing about 300 mg) was placed on a Whatman paper No. 1. The paper was then placed between two glass plates and subjected to 5 kg pressure for 5 min. After squeezing, the boundaries of the surface occupied by the meat sample and the drip of meat juice were outlined on the paper and planimeterized. The forced drip size of meat juice was measured as the difference between both surfaces. Based on the obtained value, water absorption (cm^2^) of meat was interpreted (a higher value indicates lower water absorption).

Thawing (free) drip was determined in the thawed meat samples. It was calculated based on the weight of plasma drip, in comparison to that of the sample (precision: 0.01 g). The value was expressed as drip percentage.

(2) $$\mathrm{Wr}\;(\%)=\frac{MI-MII}{MI}\;\times \;100\%$$

where Wr is the size of thawing drip (%), *MI* is the sample weight before thawing (g), and *MII* is the sample weight after thawing (g).

Shear force was measured in raw meat samples using a TA texture meter (XT plus; Stable Micro System Ltd., Surrey, UK). Briefly, the samples were cut into cylinders (along muscle fibers) of 1.0 cm diameter using a cork borer and sliced using a Warner–Bratzler blade with a triangular notch. The shear force required to cut them (N/cm^2^) was recorded, and the mean values of three successive replications (almost similar values) were determined.

For textural analysis, samples from each batch of raw meat were cut into cubes with sides of 20 mm. Their texture parameters were determined by texture profile analysis using CT3-25 texture analyzer (Brookfield, WI, USA) equipped with a cylindrical attachment (diameter: 38.1 mm, length: 20 mm). Each sample was compressed twice and reduced to 50% of its height with a roll travel speed of 2 mm/s, with a 2-s interval between compressions. Using Texture Pro CT software (V.1.9 Build 39; Brookfield, WI, USA), the following texture parameters were determined in the samples: hardness 1 and 2, stiffness up to 5 and 8 mm, adhesiveness, resilience, cohesiveness, springiness, chewiness, and gumminess. All these parameters were counted automatically during serial measurements.

### 2.3. Sensory Evaluation

The sensory properties of marinated horse meat samples were evaluated as described by Baryłko-Pikielna and Matuszewska [49]. Briefly, 100 g of samples was steamed at 95 °C until their internal temperature reached 80 °C ± 2 °C as determined using a digital thermometer with a needle probe (Sous Vide Thermapen; MERA, Warsaw, Poland). Before sensory evaluation, the samples were cooled down to 20 °C ± 2 °C and cut perpendicular to the fibers into 1.5-cm-thick slices. The slices were kept in disposable plastic boxes containing lids, individually coded, and offered in a random order to the evaluation panel consisting of six members (3 males and 3 females, aged 26–46 years). The members were experienced in evaluating meat and its products. Each sample was assessed in triplicate by the panel. Sensitivity and sensory fitness of the samples were tested in accordance with the ISO, 8586-2:2008 [50] and ISO, 8587:2006 standards [51]. Qualitative indices of the samples were assessed using a 5-point scale as follows: intensity of aroma (5 = very strong, 1 = negative and very poorly perceptible), intensity of taste (5 = very strong, 1 = negative and very poorly perceptible), desirability of aroma (5 = highly desirable, 1 = not desirable), desirability of taste (5 = highly desirable, 1 = not desirable), juiciness (5 = very juicy, 1 = very dry), and tenderness (5 = very tender, 1 = very hard). The evaluation was conducted in a specific laboratory that met the relevant standard requirements [52]. Before testing each sample, the evaluators took a 30-s break and washed their mouths with mineral water. The evaluation was conducted in 10 sessions, and 17 samples were assessed in each.

### 2.4. Statistical Analysis

All parameters were measured and sensory characteristics were assessed in triplicate. The results were statistically analyzed after grouping. All the observations (6 selected substances used for marination × 12 batches × 2 age groups × 2 storage times) were considered in the statistical analysis. Selected physical and chemical properties, texture, and sensory attributes of meat samples were analyzed by a three-way analysis of variance (ANOVA), using the GLM procedure in Statistica (STATISTICA v. 10; StatSoft, Krakow, Poland). The substances selected for marinating, age group of the animals, and period of storage were considered as a fixed effect and batch was considered as a random effect in the analyses. In the model, batch was included as a sensory variable (selected substances × age group × storage time) together with the main effects and their interaction, as well as the panelist included in the sensory evaluation. The significance of the main effects and their interaction was tested using batch as the error term (selected substances × age group × storage time). If the effects were found to be significant (*p* < 0.05), the means were compared using post hoc Tukey’s honestly significant difference test (ANOVA).

## 3. Results and Discussion

The results obtained from the chemical composition analysis of the horse meat samples are presented in Table 1. It was observed that the fat content of meat was statistically significantly influenced by the age of the horses. The meat samples obtained from the carcasses of older horses had a higher amount of fat (*p* < 0.05). A moderate amount of protein was also found in the meat of older horses, but the differences were statistically insignificant. This is in line with the study of Znamirowska [53], which reported that the fat content in horse meat increased with the age of animals. The authors observed that in foals (horses up to 2 years of age), the level of fat (2.37%) and protein (20.04%) was the lowest, while the content of water was the highest (76.42%) [53]. As the animals aged, the proportions of individual components changed, and in horses aged 2–7 years, the values were 3.46%, 21.41%, and 74.04%, respectively. In the case of horses from 7- to 12-year and 12- to 17-year age groups, a further increase in fat and protein content, and a decrease in water content in meat were noted. In the group of the oldest horses (over 17 years), the levels of fat, protein, and water were 5.36%, 22.38%, and 71.03%, respectively [53]. In addition, according to Korzeniowski et al. [54], as the age of horses increases, the meat retains less water, and more fat and minerals.

The present study showed no significant effect of increasing the time of freezer storage on the basic chemical composition of horse meat. The results confirmed those of previous research works [37,38], which also did not show any significant effect of frozen storage on the chemical composition of horse meat.

The results obtained from the analysis of the selected physical and chemical characteristics of horse meat samples are presented in Table 2. It was observed that the pH of the meat samples was statistically significantly influenced by the type of treatment applied. Marinating with malic acid decreased the pH of the meat obtained from the carcasses of young horses (*p* < 0.05). A similar effect was found when phosphates were added to malic acid- and lactic acid-marinated meat samples. The interaction between frozen storage and treatment type, in turn, influenced the acidity of the meat obtained from older horses. Marinating with phosphates with salt and phosphates with rosemary significantly increased the pH of the meat from older horses (*p* < 0.05). This is in line with the study of Bianchi et al. [55], which showed that the marination of turkey breast meat with a solution containing 2.0% sodium tripolyphosphate (STPP) and 1.4% sodium chloride caused a 0.20 unit increase in the pH of the material. Similarly, Mudalal et al. [29] observed that when chicken breast fillets were marinated with a solution of 0.3% STPP, their pH increased by 0.15 units. In turn, Garner et al. [56] marinated fresh meat from chicken breasts with 0.25% and 0.5% STPP solutions, and observed an increase in pH (*p* < 0.05) in both fresh meat and meat subjected to frozen storage at −20 °C for 6 days after STPP addition, which was attributed to marination with STPP solutions. Khan et al. [57] marinated duck breast meat samples with a 1.5% STPP solution and 3% salt, and noted an increase in pH in comparison to the control. When the marinating time was increased to 7 days, the pH of the meat samples increased further.

It was observed that the interaction between age, storage period, and type of treatment had a statistically significant influence on the color parameter a* of the meat sample. A lower proportion of red color was found in the samples marinated with lactic and malic acids, while a higher proportion was found in phosphate with salt-marinated samples of meat from young horses, after 1 month of frozen storage (*p* < 0.05). In the case of meat from older horses, a statistically significant increase in red color was noted in the samples marinated with phosphates (with salt and rosemary). It should be emphasized that phosphates, and mainly polyphosphates, moderately protect myoglobin against oxidation by sequestering iron and copper ions, and thus contribute to preserving the red color of fresh meat, as well as the pink color of marinated meat. The results of our previous studies showed that the color parameter a* decreased during cold storage in horse meat marinated with acid solutions (*p* < 0.05) [39].

Similarly to parameter a*, the interaction between age, storage period, and type of treatment also had an influence on parameter b* (*p* < 0.05). After 3 months of frozen storage, a higher (*p* < 0.05) proportion of yellow color was observed in the lactic acid-marinated samples of meat from young horses, in comparison to the control sample. However, in the case of meat samples from older horses, a decrease in parameter b* was observed after a 3-month freezer storage period if lactic acid, phosphates, and salt were applied to the marinade, and phosphates were added after marination with lactic acid, in comparison to the control sample (*p* < 0.05). In the same period of frozen storage, the proportion of yellow color was found to be increased in the control samples of meat obtained from older horses (*p* < 0.05).

Mudalal et al. [29] found an increase in brightness, and the color parameters a* and b* in chicken breast meat samples marinated with 0.3% STPP, in comparison to the control samples. In turn, Bianchi et al. [55] observed an increase in brightness and color parameter b*, but a decrease in parameter a* in turkey breast meat samples marinated with a solution containing 2.0% STPP and 1.4% sodium chloride, in comparison to non-marinated meat samples. Garner et al. [56] marinated fresh meat from chicken breasts with 0.25% and 0.5% STPP solutions, and noted a decrease in brightness and a* as well as b* parameter in the meat samples. In the case of meat subjected to frozen storage for 6 days at a temperature of −20 °C after the addition of STPP, a decrease in brightness, as well as both a* and b* parameters, was observed [56].

The results showed that the type of treatment had a statistically significant influence on the hydration properties of horse meat. Compared to the control sample, higher (*p* < 0.05) thermal and forced drips were observed in the meat samples from the carcasses of young as well as older horses in each frozen storage period with most of the marinating substances used (except for the samples marinated with phosphates with salt and those marinated with phosphates with rosemary, in which the differences in forced leakage were mostly statistically insignificant).

Phosphates have the ability to buffer meat and change the pH of meat proteins from the isoelectric point, thus contributing to improving their water-holding properties [56]. Furthermore, these substances can open the structure of proteins. Such “open” muscle proteins exhibit a greater water-binding capacity, which explains the better water retention of meat observed during heat treatment. In addition, phosphates reduce muscle contractility during thermal treatment, and hence increase the efficiency of the process.

The thermal drip values of the analyzed meat samples were found to be statistically significantly influenced by the interaction between the storage period and the treatment applied, as well as by the interaction between the age of the horses and the treatment.

A higher thermal drip was observed in the samples of meat from young horses after 3 months of frozen storage (except for those marinated with phosphates). In general, the values of thermal and thawing drip were found to be higher in the meat of young horses in all the storage periods, regardless of the type of treatment. However, the level of thawing leakage was found to be statistically significantly influenced by the type of treatment, and the interaction effect between age and the treatment applied. In general, thawing leakage was higher (*p* < 0.05) in all the tested meat samples compared to the control. However, higher (*p* < 0.05) thawing leakage was noted in the meat of young and old horses marinated with acid solutions, and, after 3 months of freezer storage, in the meat from young horses marinated with phosphates with rosemary and both types of acids with phosphates. A similar relationship has been reported in our earlier studies [37].

Marinating with different compounds is carried out to improve the hydration properties of meat. For instance, Pérez-Chabela et al. [41] analyzed the hydration parameters of horse meat marinated with CaCl_2_ and observed an increase in their values. By contrast, Pérez et al. [42] observed a decrease in water retention capacity in all the groups of meat samples marinated with CaCl_2_, and reported that this effect was related to the proteolysis of myofibrillar proteins and the decrease in the pH value. Similarly, Aktaş et al. [33] found a statistically significant increase in thermal drip values in beef samples marinated with CaCl_2_ and NaCl solutions.

The results obtained from the texture analysis of the horse meat samples are presented in Table 3. It was observed that the shear force of meat was significantly influenced by the age of the horses. Regardless of the frozen storage period, the force needed to cut was lower (*p* < 0.05) in the samples of meat obtained from the carcasses of young horses. Taking into account the type of substance used for marinating the meat samples, it was noted that the phosphate–rosemary solution caused a statistically significant increase in shear force, and, as a result, a higher force was needed to cut the meat obtained from the carcasses of older horses after 3 months of frozen storage, in comparison to the control sample.

Qin et al. [58] marinated beef samples with 5% disodium polyphosphate, 3% trisodium polyphosphate, 3% sodium hexametaphosphate, and 3% STPP, and investigated the effect of these substances on the shear force values of the material. They found that polyphosphates significantly reduced the shear force of the samples, as compared to the control. Similarly, Wang and Tang [59] marinated beef samples for 6 h with a 0.5% malic acid solution and found that the shear force values of the samples were significantly decreased in comparison to the control.

The present study revealed that the age of horses had a statistically significant influence on the texture parameters of the meat samples. The results showed that the values of hardness, stiffness, gumminess, and chewiness of the meat increased with the animal’s age (*p* < 0.05). Additionally, the interaction between storage time and treatment statistically significantly influenced the values of hardness 1, while the interaction between age and storage time significantly influenced the values of stiffness up to 5 mm. The latter parameter was also found to be significantly influenced by the interaction between all the analyzed factors.

Previous studies in different species [34,36,60,61] have indicated that marinating with organic acids reduced the hardness of meat. Hosseini and Esfahani Mehr [34] showed that organic acids decreased the pH value of beef meat samples, thereby leading to the solubilization of collagen tissue and causing an increase in the tenderness of the material.

Different compounds have been used for marinating horse meat, to achieve improved texture parameters. Studies [43] on similar material showed that CaCl_2_ solution used for marinating contributed to lowering the hardness of meat samples in comparison to the control samples.

The results obtained from the sensory evaluation of the horse meat samples are presented in Table 4. It was noted that the interaction between age and the treatment procedure statistically significantly influenced the tenderness and juiciness of the meat samples. In the case of meat obtained from older horses, a statistically significant difference was noted after 3 months of frozen storage in these parameters, between the control sample and samples marinated with phosphate solutions with salt. Similarly, a statistically significant difference in tenderness and juiciness was found between the control sample and the samples treated with lactic acid with phosphates, and malic acid with phosphates, after 1 month of storage in freezing conditions. An improvement in tenderness was observed during sensory evaluation in meat samples marinated with lactic acid with phosphates and malic acid with phosphates (*p* < 0.05). Another study of our research group [40] showed that substances such as citric acid, and 0.2 and 0.3 M CaCl_2_ caused a statistically significant improvement in meat tenderness.

Phosphates are applied as functional additives in meat processing to improve the sensory quality of the raw material (tenderness, juiciness, color, aroma) [30]. Capita et al. [62] showed that chicken legs immersed for 15 min in 10% and 12% solutions of TSP showed improved sensory quality compared to the control sample. Dipping in a 10% TSP solution contributed to improving the smell and color of chicken legs, while dipping in a 12% TSP solution enhanced the color and overall acceptability. Sheard et al. [63] injected polyphosphate solutions into pork, and investigated their effect on the juiciness and tenderness of the meat. They analyzed the samples after cooking by grilling to achieve a temperature of 72.5 °C or 80.0 °C in the geometric center of the product. The effect of two doses of injection (5% and 10%) and three concentrations of STPP (0%, 3%, and 5%) were studied. The results of the sensory evaluation showed that pork steaks injected with a solution of 5% STPP at a dose of 10%, and cooked to a temperature of 80 °C exhibited better tenderness, but the juiciness of the meat remained unchanged.

## 4. Conclusions

The use of malic acid and malic acid with the subsequent addition of phosphates for marinating meat, in order to lower its pH value, is particularly recommended for the raw material obtained from carcasses of young horses. On the other hand, for horse meat obtained from carcasses of older animals, the use of phosphates is advisable, as it allows the pH value of the meat to be increased. With the age of horses, the values of cutting force, hardness, stiffness, gumminess, and chewiness of the meat increase (*p* < 0.05). This is most likely caused by the increase in the amount of collagen and its cross-linking in the meat of older horses. The present study showed that the application of lactic acid and malic acid for marinating the meat of young horses caused a decrease in the proportion of red color and an increase in the proportion of yellow color, especially after 3 months of frozen storage. In turn, an increase in the value of the a* parameter and most often a decrease in the b* parameter were observed with the use of phosphates for marinating meat from the carcasses of older horses. Each of the substances used for marination caused a decline in the hydration properties of horse meat. However, the lowest values of forced and thermal leakage from meat were achieved with the use of phosphates and salt.

## Figures and Tables

**Table 1:** Chemical composition of the tested horse meat samples (from each age group: 12 carcasses × 2 parts of muscle samples × 7 steaks = 168 samples of steaks) (%, x ± SE).

Specification	Months	Age	Control Sample	Lactic Acid	Malic Acid	Phosphates with Salt	Phosphates with Rosemary	Lactic Acid with Phosphates	Malic Acid with Phosphates	ANO VA
Protein (%)	1	Y O	19.83 ± 0.86 20.50 ± 0.44	19.53 ± 1.03 20.53 ± 1.46	19.37 ± 0.21 20.43 ± 0.12	20.30 ± 0.40 20.60 ± 0.00	19.83 ± 0.40 20.13 ± 0.31	19.43 ± 0.40 20.07 ± 0.76	19.07 ± 0.50 20.40 ± 0.46	
	3	Y O	20.13 ± 0.05 21.03 ± 0.06	19.90 ± 0.00 20.50 ± 0.17	20.02 ± 0.12 20.67 ± 0.67	20.08 ± 0.20 20.13 ± 0.31	19.67 ± 0.29 20.50 ± 0.10	20.10 ± 0.10 20.60 ± 0.56	19.93 ± 0.47 20.23 ± 0.32	
Fat (%)	1	Y O	4.17 ^x^ ± 1.76 8.10 ^y^ ± 2.26	4.40 ^x^ ± 1.03 10.80 ^y^ ± 2.86	4.43 ± 0.52 4.67 ± 1.30	3.80 ± 0.95 4.83 ± 0.85	3.43 ± 0.06 5.20 ± 0.95	4.10 ± 0.35 4.13 ± 1.06	4.30 ± 1.54 5.23 ± 1.05	A *
	3	Y O	1.47 ^x^ ± 0.10 5.40 ^y^ ± 0.15	4.70 ^x^ ± 0.12 6.07 ^y^ ± 0.96	1.20 ^x^ ± 0.15 2 77 ^y^ ± 0.10	4.40 ± 0.10 4.97 ± 0.75	3.17 ± 0.59 4.20 ± 0.10	1.60 ^x^ ± 0.10 4.50 ^y^ ± 0.85	2.53 ± 1.99 2.77 ± 1.72	
Water (%)	1	Y O	74.20 ± 1.47 70.23 ^a^ ± 2.15	73.63 ^x^ ± 2.29 67.20 ^y,c^ ± 4.29	74.90 ± 0.72 73.37 ± 1.92	74.00 ± 2.04 73.40 ± 0.69	74.70 ± 0.10 73.07 ± 0.81	74.50 ± 2.23 75.43 ^b^ ± 0.55	75.10 ± 1.15 73.17 ± 0.67	
	3	Y O	76.30 ± 0.10 72.80 ± 0.10	73.40 ± 1.14 72.40 ^d^ ± 0.10	75.03 ± 0.46 74.20 ± 0.20	74.27 ± 0.81 73.80 ± 0.20	74.90 ± 0.52 74.10 ± 0.20	76.93 ± 1.35 74.20 ± 0.20	75.70 ± 2.87 74.23 ± 1.53	

Notes: ^a,b^ different letters in the same row indicate statistically significant differences between the control sample and the samples marinated with selected substances (*p* < 0.05). ^x,y^ Different letters in the same column indicate statistically significant differences between the age groups of horses in particular periods of frozen storage (*p* < 0.05). ^c,d^ Different letters in the same column indicate statistically significant differences between the periods of frozen storage in particular age groups of horses (*p* < 0.05). ANOVA: three-way ANOVA; A, age of animal. * *p* < 0.05. Y and O denote younger and older horses, respectively.

**Table 2:** Selected physical and chemical properties of the tested horse meat samples (from each age group: 12 carcasses × 2 parts of muscle samples × 7 steaks = 168 samples of steaks) (%, x ± SE).

Specification	Months	Age	Control Sample	Lactic Acid	Malic Acid	Phosphates with Salt	Phosphates with Rosemary	Lactic Acid with Phosphates	Malic Acid with Phosphates	ANOVA
pH	1	Y O	5.55 ^a^ ± 0.02 5.46 ± 0.04	5.32 ± 0.02 5.27 ± 0.09	5.20 ^b^ ± 0.05 5.29 ± 0.09	5.60 ± 0.03 5.55 ± 0.08	5.63 ± 0.06 5.58 ± 0.02	5.26 ^b^ ± 0.14 5.37 ± 0.04	5.20 ^b^ ± 0.21 5.18 ± 0.11	T * S × T *
	3	Y O	5.81 ^a^ ± 0.13 5.59 ^a^ ± 0.05	5.45 ^b^ ± 0.03 5.36 ± 0.14	5.32 ^b^ ± 0.12 5.38 ± 0.06	5.80 ± 0.06 5.89 ^b^ ± 0.12	5.70 ± 0.13 5.89 ^b^ ± 0.03	5.53 ± 0.10 5.46 ± 0.04	5.31 ^b^ ± 0.03 5.42 ± 0.10	
L *	1	Y O	39.34 ± 3.62 36.18 ± 0.96	40.93 ± 2.42 33.33 ± 2.74	32.94 ± 2.11 41.28 ± 3.63	41.27 ± 0.89 39.89 ± 2.99	41.00 ± 2.46 40.57 ± 3.43	36.94 ± 3.06 43.03 ± 2.06	43.16 ± 2.59 40.68 ± 3.91	
	3	Y O	38.36 ± 4.71 43.47 ± 3.50	42.51 ± 2.65 38.86 ± 0.71	39.32 ± 3.55 40.65 ± 6.85	39.72 ± 2.37 40.80 ± 1.35	40.52 ± 3.01 41.43 ± 1.86	41.27 ± 1.56 34.33 ± 0.93	41.12 ± 2.90 42.01 ± 4.02	
a *	1	Y O	15.18 ^a^ ± 2.15 13.49 ^a,^^c^ ± 3.96	11.74 ^b^ ± 0.60 8.16 ^b^ ± 0.85	7.27 ^b^ ± 0.55 9.62 ± 3.34	26.28 ^b,^^c^± 1.53 17.65 ^b^ ± 1.22	17.19 ± 1.19 17.45 ^b^ ± 0.86	12.25 ± 0.95 12.12 ± 2.21	11.92 ± 1.13 9.33 ± 0.47	A × S × T *
	3	Y O	13.35 ^x^ ± 2.22 7.99 ^a,y,^^d^ ± 1.21	8.68 ± 0.93 7.16 ± 0.75	9.92 ± 0.47 6.96 ± 0.43	11.05 ^x,d^± 2.34 18.73 ^b,y^ ± 0.21	17.98 ± 0.89 15.08 ^b^ ± 0.74	9.79 ± 0.24 8.56 ± 0.19	10.22 ± 2.00 8.38 ± 0.73	
b *	1	Y O	6.83 ± 0.77 7.24 ^c^ ± 0.86	8.43 ± 0.22 5.72 ± 0.16	6.84 ± 0.53 8.69 ± 1.30	7.08 ± 1.09 6.61 ± 0.44	6.35 ± 0.72 7.90 ± 0.88	9.13 ± 1.04 10.16 ± 1.59	10.06 ± 1.64 8.97 ± 0.40	A × S × T *
	3	Y O	6.94 ^a,x^ ± 0.76 12.59 ^a,y,d^ ± 2.12	10.75 ^b^ ± 2.27 8.73 ^b^ ± 1.36	8.65 ± 0.70 10.29 ± 1.17	8.06 ± 0.33 7.59 ^b^ ± 0.22	7.00 ± 0.70 9.08 ± 1.56	9.95 ± 0.84 6.83 ^b^ ± 0.10	9.27 ± 2.03 9.22 ± 1.43	
Thermal drip (%)	1	Y O	20.00 ^a,c^ ± 0.30 19.70 ^a^ ± 1.50	32.80 ^b,x,^^c^± 2.00 27.10 ^b,y^ ± 1.00	33.30 ^b,^^c^± 0.15 29.85 ^b^ ± 1.00	25.25 ^b^ ± 1.50 23.50 ^b^ ± 2.00	24.80 ^b^ ± 0.20 21.75 ± 1.00	34.35 ^b,^^c^± 3.00 30.95 ^c^ ± 1.00	34.75 ^b^ ± 2.00 32.05 ^b^ ± 2.00	T * S × T * A × T *
	3	Y O	29.75 ^a,x,^^d^± 1.00 21.35 ^a,y^ ± 0.25	34.50 ^x,^^d^ ± 1.00 25.95 ^b,y^ ± 0.50	38.90 ^b,^^d^± 0.10 35.90 ^b^ ± 0.90	23.25 ± 0.25 22.30 ^b^ ± 0.30	32.05 ^b^ ± 0.30 25.30 ^b^ ± 0.30	35.45 ^b,^^d^ ± 1.00 33.25 ^b,^^d^± 0.05	36.45 ^b,x^ ± 1.00 31.75 ^y^ ± 0.25	
Forced drip (cm^2^)	1	Y O	6.35 ^a^ ± 0.10 3.70 ^a^ ± 0.35	9.25 ^b^ ± 1.25 9.32 ^b^ ± 0.26	11.17 ^b^ ± 1.25 8.45 ^c^ ± 0.75	6.70 ± 0.20 6.00 ± 0.20	6.40 ± 1.20 4.85 ± 0.05	10.63 ^b^ ± 1.05 10.60 ^b^ ± 0.10	11.56 ^b^ ± 0.73 10.65 ^b^ ± 0.65	T *
	3	Y O	7.03 ^a^ ± 0.10 4.10 ^a^ ± 1.88	8.80 ^b^ ± 0.20 9.50 ^b^ ± 2.95	12.93 ^b^ ± 0.20 11.60 ^b,^^d^± 2.02	9.30 ^b^ ± 0.15 7.00 ± 3.90	7.70 ± 0.20 6.60 ± 0.70	12.20 ^b,x^ ± 0.20 7.98 ^y^ ± 0.18	10.88 ^b^ ± 0.53 9.20 ^b^ ± 2.66	
Thawing drip (%)	1	Y O	3.19 ^a^ ± 0.23 2.65 ^a^ ± 0.53	13.42 ^b^ ± 0.65 13.27 ^b^ ± 1.03	14.24 ^b^ ± 0.59 12.54 ^b^ ± 0.56	9.62 ± 0.88 9.40 ± 0.52	9.19 ± 0.32 8.92 ± 0.76	9.79 ^x^ ± 0.79 2.67 ^y^ ± 0.20	9.88 ± 0.33 7.28 ± 0.59	T * A × T *
	3	Y O	8.65 ^a,x^ ± 0.37 4.38 ^a,y^ ± 0.65	15.58 ^b^ ± 0.87 14.74 ^b^ ± 0.98	21.42 ^b,x^ ± 1.02 14.63 ^b,y^ ± 0.54	12.10 ± 0.49 11.70 ^b^ ± 1.01	13.39 ^b^ ± 1.76 11.83 ± 0.56	17.95 ^b,x^ ± 1.03 9.79 ^y^ ± 0.42	20.37 ^b,x^ ± 1.02 15.56 ^b,y^ ± 0.99	

Notes: ^a,b^ different letters in the same row indicate statistically significant differences between the control sample and the samples marinated with selected substances (*p* < 0.05). ^x,y^ Different letters in the same column indicate statistically significant differences between the age groups of horses in particular periods of frozen storage (*p* < 0.05). ^c,d^ Different letters in the same column indicate statistically significant differences between the periods of frozen storage in particular age groups of horses (*p* < 0.05). ANOVA, three-way ANOVA; S, selected substances; A; age of animal; T, time of storage. * *p* < 0.05. Y and O denote younger and older horses, respectively.

**Table 3:** Texture parameters of the tested horse meat samples (from each age group: 12 carcasses × 2 parts of muscle samples × 7 steaks = 168 samples of steaks) (%, x ± SE).

Specification	Months	Age	Control Sample	Lactic Acid	Malic Acid	Phosphates with Salt	Phosphates with Rosemary	Lactic Acid with Phosphates	Malic Acid with Phosphates	ANOVA
Shear force (N/cm^2^)	1	Y O	75.21 ^x^ ± 6.53 79.78 ^y^ ± 6.19	70.63 ± 9.46 72.26 ± 5.75	62.78 ^x^ ± 9.66 72.26 ^y^ ± 9.68	59.84 ^x^ ± 7.85 82.40 ^y^ ± 7.66	83.05 ± 10.80 84.03 ± 9.32	71.28 ^x^ ± 7.23 85.02 ^y^ ± 10.76	66.38 ^x^ ± 7.40 83.71 ^y^ ± 4.42	A *
	3	Y O	75.20 ^x^ ± 3.54 78.48 ^a,y^ ± 8.46	59.83 ^x^ ± 6.13 76.19 ^y^ ± 2.83	78.80 ^x^ ± 4.84 81.74 ^y^ ± 3.61	68.99 ^x^ ± 6.89 81.42 ^y^ ± 3.54	86.00 ^x^ ± 4.30 107.25 ^b,y^ ± 5.03	83.38 ^x^ ± 3.53 92.21 ^y^ ± 4.50	74.55 ^x^ ± 2.19 79.13 ^y^ ± 8.03	
Hardness 1 (N)	1	Y O	144.35 ^a,x^ ± 4.04 226.06 ^y^ ± 6.42	75.74 ^b,x^ ± 7.93 173.39 ^y^ ± 3.70	83.43 ^b,x^ ± 8.72 161.83 ^y^ ± 4.14	88.01 ^b,x^ ± 5.56 153.50 ^y^ ± 9.27	107.80 ^x^ ± 11.68 194.60 ^y^ ± 9.45	99.93 ^x^ ± 3.35 164.52 ^y^ ± 4.64	138.11 ^x,c^ ± 7.09 237.55 ^y,c^ ± 3.86	A * S × T *
	3	Y O	84.04 ^a,x^ ± 4.96 161.44 ^y^ ± 1.79	65.22 ^b,x^ ± 1.98 67.55 ^y^ ± 7.86	66.69 ^b,x^ ± 4.32 220.88 ^y^ ± 5.53	125.69 ^b,x^ ± 4.77 155.05 ^y^ ± 5.57	108.15 ^x^ ± 5.42 136.29 ^y^ ± 7.28	86.04 ^x^ ± 2.60 119.79 ^y^ ± 1.36	11.97 ^b,x,d^ ± 3.30 23.06 ^y,d^ ± 2.15	
Hardness 2 (N)	1	Y O	89.69 ^x^ ± 8.18 98.55 ^y^ ± 6.49	45.72 ^x^ ± 2.08 109.50 ^y^ ± 5.98	56.52 ^x^ ± 2.84 91.05 ^y^ ± 7.26	61.04 ^x^ ± 1.54 100.06 ^y^ ± 3.51	67.64 ^x^ ± 1.99 106.60 ^y^ ± 4.51	55.32 ^x^ ± 4.62 101.30 ^y^ ± 7.35	87.99 ^x^ ± 8.70 150.14 ^y,c^ ± 8.98	A *
	3	Y O	51.96 ^a,x^ ± 4.05 113.75 ^a,y^ ± 4.74	45.75 ± 2.81 48.71 ± 2.23	44.39 ^b,x^ ± 0.16 120.83 ^y^ ± 12.86	55.54 ^x^ ± 5.93 100.94 ^y^ ± 9.21	55.81 ^x^ ± 2.17 79.66 ^y^ ± 5.51	65.15 ^x^ ± 1.31 72.67 ^y^ ± 5.37	9.07 ^b,x^ ± 1.86 16.96 ^b,y,d^ ± 3.37	
Stiffness up to 5 mm (N)	1	Y O	19.48 ^x^ ± 1.46 36.06 ^y,c^ ± 3.85	11.68 ^x^ ± 2.59 23.05 ^y^ ± 1.60	8.17 ^x^ ± 1.16 26.62 ^y^ ± 0.43	9.84 ^x^ ± 0.89 14.81 ^y^ ± 1.84	10.45 ^x^ ± 1.87 42.78 ^y,c^ ± 1.03	13.67 ± 1.47 16.99 ± 2.00	14.86 ± 1.74 15.42 ± 1.64	A * A × S *
	3	Y O	5.87 ^x^ ± 1.86 9.62 ^y,d^ ± 0.04	5.28 ^x^ ± 1.85 9.24 ^y^ ± 1.00	4.60 ^x^ ± 1.88 10.89 ^y^ ± 0.24	12.05 ^x^ ± 1.60 16.34 ^y^ ± 1.57	8.26 ± 1.98 9.95 ^d^ ± 0.64	7.99 ^x^ ± 1.96 16.89 ^y^ ± 1.08	1.65 ^x^ ± 0.16 3.31 ^y^ ± 0.32	
Stiffness up to 8 mm (N)	1	Y O	92.53 ^a,x^ ± 13.13 180.62 ^a,y,c^ ± 24.18	38.93 ^b,x^ ± 4.50 66.39 ^b,y^ ± 7.96	33.48 ^b,x^ ± 5.98 114.18 ^y^ ± 2.19	40.90 ^b,x^ ± 8.05 82.66 ^b,y^ ± 3.82	48.97 ^b,x^ ± 2.55 163.25 ^y,c^ ± 5.85	64.23 ^b,x^ ± 1.90 79.09 ^b,y^ ± 5.53	63.58 ^b,x^ ± 2.49 82.61 ^b,y^ ± 1.37	A * A × S × T *
	3	Y O	27.09 ^x^ ± 4.90 54.10 ^y,d^ ± 0.42	20.95 ^x^ ± 2.46 32.01 ^y^ ± 3.49	22.69 ^x^ ± 1.01 67.23 ^y^ ± 1.80	73.28 ^x^ ± 5.31 80.89 ^y^ ± 5.36	43.19 ^x^ ± 4.50 62.87 ^y,d^ ± 1.19	36.23 ^x^ ± 1.30 64.77 ^y^ ± 3.91	2.85 ^x^ ± 0.25 10.85 ^y^ ± 1.28	
Adhesiveness (mJ)	1	Y O	4.90 ± 1.10 3.10 ± 0.27	1.60 ± 1.03 1.30 ± 0.05	1.10 ± 0.07 1.50 ± 0.05	4.47 ± 0.67 2.47 ± 0.80	0.90 ± 0.07 5.50 ± 0.71	1.05 ± 0.09 2.90 ± 0.09	0.95 ± 0.05 1.60 ± 0.07	
	3	Y O	4.20 ± 0.68 5.20 ± 0.37	0.40 ± 0.14 0.40 ± 0.01	2.75 ± 0.35 0.33 ± 0.06	4.53 ± 0.90 0.90 ± 0.09	2.20 ± 0.67 0.80 ± 0.02	2.55 ± 0.18 0.45 ± 0.07	0.40 ± 0.14 0.40 ± 0.01	
Resilience	1	Y O	0.05 ± 0.01 0.11 ± 0.01	0.09 ± 0.04 0.12 ± 0.01	0.08 ± 0.01 0.13 ± 0.02	0.10 ± 0.04 0.16 ± 0.04	0.14 ± 0.07 0.08 ± 0.01	0.11 ± 0.03 0.11 ± 0.03	0.12 ± 0.03 0.11 ± 0.01	
	3	Y O	0.14 ± 0.02 0.12 ± 0.01	0.18 ± 0.02 0.17 ± 0.01	0.07 ± 0.01 0.09 ± 0.01	0.12 ± 0.01 0.08 ± 0.01	0.12 ± 0.01 0.14 ± 0.02	0.12 ± 0.04 0.20 ± 0.01	0.14 ± 0.02 0.11 ± 0.01	
Cohesiveness	1	Y O	0.07 ± 0.01 0.17 ± 0.01	0.20 ± 0.01 0.21 ± 0.02	0.14 ± 0.01 0.31 ± 0.01	0.21 ± 0.02 0.25 ± 0.05	0.25 ± 0.01 0.10 ± 0.04	0.21 ± 0.03 0.19 ± 0.07	0.24 ± 0.01 0.25 ± 0.02	
	3	Y O	0.28 ± 0.01 0.25 ± 0.02	0.33 ± 0.05 0.39 ± 0.04	0.26 ± 0.08 0.23 ± 0.03	0.19 ± 0.08 0.15 ± 0.01	0.20 ± 0.04 0.20 ± 0.02	0.24 ± 0.01 0.40 ± 0.06	0.29 ± 0.02 0.29 ± 0.05	
Springiness (mm)	1	Y O	2.25 ± 0.06 4.10 ± 0.74	3.39 ± 0.08 3.54 ± 0.05	3.41 ± 0.05 3.84 ± 0.55	3.13 ± 0.28 3.47 ± 0.07	3.50 ± 0.38 2.91 ± 0.64	3.97 ± 0.26 3.47 ± 0.48	4.02 ± 0.36 3.48 ± 0.28	
	3	Y O	3.36 ± 0.07 2.70 ± 0.01	4.28 ± 0.22 3.81 ± 0.37	4.18 ± 0.60 1.75 ± 0.24	2.66 ± 0.59 3.38 ± 0.23	2.95 ± 0.05 3.54 ± 0.09	3.88 ± 0.52 4.33 ± 0.04	2.01 ± 0.06 2.77 ± 0.07	
Gumminess (mm)	1	Y O	10.14 ^x^ ± 2.58 48.43 ^y^ ± 2.31	15.14 ^x^ ± 2.77 36.41 ^y^ ± 1.67	11.69 ^x^ ± 0.64 50.16 ^y^ ± 3.14	18.48 ^x^ ± 1.87 38.37 ^y^ ± 1.01	26.29 ± 3.82 19.46 ± 2.11	20.97 ^x^ ± 3.58 31.25 ^y^ ± 3.21	33.14 ^x^ ± 3.82 59.38 ^y^ ± 1.60	A *
	3	Y O	23.53 ^x^ ± 4.40 40.36 ^y^ ± 1.75	21.52 ^x^ ± 0.83 26.34 ^y^ ± 1.10	17.33 ^x^ ± 7.96 50.80 ^y^ ± 0.13	23.88 ± 2.61 23.25 ± 0.90	21.63 ^x^ ± 3.30 27.25 ^y^ ± 3.99	20.64 ^x^ ± 3.22 47.91 ^y^ ± 3.85	3.47 ^x^ ± 0.38 6.68 ^y^ ± 0.68	
Chewiness (mJ)	1	Y O	22.73 ^x^ ± 1.39 157.56 ^y^ ± 3.05	51.35 ^x^ ± 4.52 128.89 ^y^ ± 4.99	39.82 ^x^ ± 3.32 192.64 ^y^ ± 3.65	57.83 ^x^ ± 5.51 133.16 ^y^ ± 4.74	56.62 ^x^ ± 8.69 94.32 ^y^ ± 3.02	83.30 ^x^ ± 9.72 108.46 ^y^ ± 5.56	133.24 ^x^ ± 3.54 206.66 ^y^ ± 3.76	A *
	3	Y O	79.06 ^x^ ± 26.85 108.97 ^y^ ± 7.11	92.11 ^x^ ± 4.34 100.37 ^y^ ± 13.36	72.47 ^x^ ± 5.12 88.90 ^y^ ± 5.15	63.53 ^x^ ± 8.85 78.61 ^y^ ± 8.48	63.80 ^x^ ± 9.42 96.49 ^y^ ± 7.04	80.12 ^x^ ± 6.47 207.47 ^y^ ± 5.41	6.97 ^x^ ± 1.43 18.52 ^y^ ± 2.07	

Notes: ^a,b^ different letters in the same row indicate statistically significant differences between the control sample and the samples marinated with selected substances (*p* < 0.05). ^x,y^ Different letters in the same column indicate statistically significant differences between the age groups of horses in particular periods of frozen storage (*p* < 0.05). ^c,d^ Different letters in the same column indicate statistically significant differences between the periods of frozen storage in particular age groups of horses (*p* < 0.05). ANOVA, three-way ANOVA; S, selected substances; A, age of animal; T, time of storage. * *p* < 0.05. Y and O denote younger and older horses, respectively

**Table 4:** Sensory properties of the tested horse meat samples (from each age group: 12 carcasses × 2 parts of muscle samples × 7 steaks = 168 samples of steaks) (%, x ± SE).

Specification	Months	Age	Control Sample	Lactic Acid	Malic Acid	Phosphates with Salt	Phosphates with Rosemary	Lactic Acid with Phosphates	Malic Acid with Phosphates	ANOVA
Aroma: intensity	1	Y O	3.83 ± 0.29 3.00 ± 0.50	3.33 ± 0.76 3.67 ± 0.58	3.83 ^x^ ± 0.29 2.17 ^y^ ± 0.29	4.00 ± 0.00 4.00 ± 0.00	3.67 ± 0.58 2.67 ± 0.58	2.83 ± 0.29 2.67 ± 0.76	2.83 ± 0.29 2.50 ± 0.50	
	3	Y O	3.83 ± 0.29 3.33 ± 0.58	3.50 ± 0.50 3.17 ±1.04	3.00 ± 0.87 2.67 ± 0.58	4.00 ± 0.90 3.67 ± 0.58	4.00 ± 0.90 3.83 ± 0.29	2.50 ± 0.50 3.33 ± 0.29	2.33 ± 0.58 2.00 ± 0.00	
Aroma: desirability	1	Y O	3.83 ± 0.76 3.17 ± 0.29	3.33 ± 0.58 3.67 ± 0.58	2.33 ± 1.53 2.67 ± 0.58	4.00 ± 0.00 4.17 ± 0.29	4.33 ± 0.29 3.50 ± 0.50	2.83 ± 0.76 3.33 ± 0.58	2.83 ± 0.76 3.00 ± 0.50	
	3	Y O	3.83 ± 0.29 3.33 ± 0.58	3.83 ± 0.76 3.17 ± 1.44	2.83 ± 0.76 2.67 ± 0.58	4.33 ± 0.29 3.83 ± 0.29	4.00 ± 0.90 4.17 ± 0.29	2.83 ± 0.76 3.00 ± 0.10	2.83 ± 0.76 2.00 ± 0.00	
Tenderness	1	Y O	4.00 ^a^ ± 1.00 3.00 ^a^ ± 0.50	3.67 ± 0.58 3.50 ± 0.50	2.00 ^b^ ± 1.00 1.67 ± 0.58	4.17 ± 1.04 3.33 ± 0.29	4.17 ± 0.29 3.67 ± 0.29	1.83 ^b^ ± 0.50 1.50 ^b^ ± 0.76	1.83 ^b^ ± 0.58 1.67 ^b^ ± 1.04	A × T *
	3	Y O	4.50 ^a,x^ ± 0.50 1.83 ^a,y^ ± 0.29	3.67 ± 0.29 3.17 ± 0.29	2.83 ± 1.23 2.17 ± 0.29	4.50 ± 0.29 4.33 ^b^ ± 0.50	3.67 ± 0.50 3.50 ± 0.58	3.67 ^x^ ± 0.58 1.33 ^y^ ± 0.58	1.33 ^b^ ± 0.58 1.17 ± 0.29	
Juiciness	1	Y O	3.33 ^a^ ± 0.58 2.67 ± 0.58	3.00 ± 0.10 3.00 ± 1.00	2.67 ± 0.58 1.50 ± 0.50	2.67 ± 0.76 3.67 ± 0.58	3.00 ± 0.00 3.50 ± 0.50	1.33 ^b^ ± 0.58 2.00 ± 0.50	2.00 ± 0.87 2.50 ± 0.87	A × T *
	3	Y O	4.00 ^a,x^ ± 0.90 1.67 ^a,y^ ± 0.58	3.17 ± 0.29 2.17 ± 0.58	3.17 ± 1.04 2.00 ± 0.50	3.83 ± 0.58 4.00 ^b^ ± 0.00	3.33 ± 0.29 3.17 ± 0.29	1.33 ^b^ ± 0.58 2.67 ± 0.58	1.33 ^b^ ± 0.58 1.00 ± 0.10	
Taste: intensity	1	Y O	3.67 ± 0.58 3.33 ± 0.58	3.50 ± 0.50 3.00 ± 0.50	3.00 ± 1.73 3.00 ± 1.73	3.33 ± 0.58 4.33 ± 0.29	3.67 ± 0.58 3.67 ± 0.58	2.00 ± 1.00 2.33 ± 0.76	2.00 ± 1.00 2.33 ± 1.15	
	3	Y O	4.17 ^a^ ± 0.29 2.00 ± 1.00	3.50 ± 0.00 3.17 ± 0.58	3.00 ± 1.00 2.17 ± 1.04	4.17 ± 0.29 4.33 ± 0.58	3.33 ± 0.76 3.67 ± 0.29	1.67 ^b^ ± 1.15 3.50 ± 0.50	1.33 ^b^ ± 0.58 1.33 ± 0.58	
Taste: desirability	1	Y O	3.67 ± 0.58 3.67 ± 0.58	3.50 ± 0.50 3.17 ± 0.58	2.00 ± 1.00 1.50 ± 0.87	3.33 ± 0.58 4.33 ± 0.29	3.83 ± 0.76 3.83 ± 0.29	2.00 ± 1.00 2.50 ± 0.87	2.33 ± 1.53 1.50 ± 0.87	
	3	Y O	4.17 ^a^ ± 0.29 2.00 ± 1.00	3.67 ± 0.29 3.17 ± 0.58	3.00 ± 1.00 2.17 ± 1.04	4.33 ± 0.29 4.17 ± 0.29	3.33 ± 0.76 3.83 ± 0.29	1.83 ± 1.44 3.67 ± 0.58	1.33 ^b^ ± 0.58 1.33 ± 0.58	
General acceptability	1	Y O	3.72 ± 0.57 3.14 ± 0.24	3.39 ± 0.13 3.33 ± 0.29	2.64 ± 0.89 2.08 ± 0.55	3.44 ± 0.48 4.11 ± 0.25	3.69 ± 0.32 3.56 ± 0.32	2.08 ± 0.60 2.44 ± 0.55	2.28 ± 0.68 2.28 ± 0.61	
	3	Y O	4.08 ± 0.14 2.36 ± 0.29	3.56 ± 0.27 3.00 ± 0.58	2.97 ± 0.97 2.31 ± 0.38	4.17 ± 0.25 4.08 ± 0.33	3.58 ± 0.38 3.72 ± 0.27	1.92 ± 0.80 3.31 ± 0.25	1.75 ± 0.36 1.47 ± 0.24	

Notes: ^a,b^ different letters in the same row indicate statistically significant differences between the control sample and the samples marinated with selected substances (*p* < 0.05). ^x,y^ Different letters in the same column indicate statistically significant differences between the age groups of horses in particular periods of frozen storage (*p* < 0.05). ANOVA, three-way ANOVA; A, age of animal; T, time of storage. * *p* < 0.05. Y and O denote younger and older horses, respectively.

## Data Availability

Data available at the Department of Agricultural Processing and Commodity Science at the University of Rzeszów.
